# Application of Flow Cytometry to Determine Differential Redistribution of Cytochrome c and Smac/DIABLO from Mitochondria during Cell Death Signaling

**DOI:** 10.1371/journal.pone.0042298

**Published:** 2012-07-27

**Authors:** Heling Ng, Danielle J. Smith, Phillip Nagley

**Affiliations:** Department of Biochemistry and Molecular Biology, and ARC Centre of Excellence in Structural and Functional Microbial Genomics, Monash University, Clayton, Victoria, Australia; UMASS-Amherst/Tufts University School of Medicine, United States of America

## Abstract

Mitochondrially mediated apoptosis is characterized by redistribution of proteins from mitochondria to cytoplasm following permeabilization of the outer mitochondrial membrane. We applied flow cytometry to quantify simultaneously the redistribution of two apoptogenic proteins, cytochrome c (cyt c) and Smac/DIABLO (Smac). Mammalian cells were treated with digitonin that selectively permeabilizes the plasma membrane. Following fixation, treated cells were infused successively with primary and secondary antibodies (the latter fluorescently tagged) enabling independent detection of cyt c and Smac. Digitonin-treated cells that retain cyt c or Smac in mitochondria generate strong fluorescence signals in flow cytometry. Cells in which cyt c or Smac have transited the outer mitochondrial membrane show greatly reduced fluorescence because the proteins are lost from the digitonin-permeabilized cells. Quantitative flow cytometry revealed that in 143B TK^-^ cells treated with staurosporine, cyt c and Smac exit mitochondria asymmetrically, with cyt c redistribution preceding that of Smac. However, in HeLa cells likewise treated, cyt c and Smac exit mitochondria concurrently. Under other conditions of apoptotic induction, for example, 143B TK^-^ cells treated with MT-21 (an apoptotic inducer that binds to the mitochondrial adenine nucleotide transporter), redistribution of Smac precedes that of cyt c. The various patterns of redistribution of these proteins were confirmed by immunocytochemical analysis and confocal microscopy. We conclude that flow cytometry can be employed effectively to quantify simultaneously the redistribution of cyt c and Smac from mitochondria to the cytosol. Moreover, differential redistribution of cyt c and Smac occurs under various conditions, thereby reflecting constraints on availability of these proteins to exit mitochondria after permeabilization of the outer membrane.

## Introduction

Mitochondria play a key role in apoptosis. Permeabilization of the outer mitochondrial membrane (OMM) is one of the prominent features of apoptosis, resulting in the redistribution to the cytosol of mitochondrial intermembrane space (IMS) proteins [1], [2]. The role of many of these redistributed proteins is to facilitate the downstream apoptotic signaling cascade. These proteins include cytochrome c (cyt c), Smac/DIABLO (second mitochondrial activator of caspases/direct IAP binding protein with low PI; here designated as Smac), apoptosis inducing factor (AIF), HtrA2/Omi and Endonuclease G (EndoG), each with their own particular role [3]. For example, redistribution of cyt c into the cytosol triggers formation of the apoptosome that activates procaspase-9, while Smac antagonizes inhibitor of apoptosis (IAP) proteins to enhance caspase activity [4], [5], [6], [7].

The Bcl-2 family of proteins is collectively responsible for the cellular decision of whether or not to permeabilize the OMM under signaling regimes, often due to stress, which potentially lead to apoptosis. The Bcl-2 family consists of pro-apoptotic proteins (e.g. Bax, Bak) as well as competing anti-apoptotic proteins (e.g. Bcl-2 itself, Bcl-x_L_) [8]. Although the exact mechanism of how such permeabilization occurs in the OMM has not been elucidated, current notions embrace the possibilities of relatively non-specific pores or channels that form in the OMM to allow efflux of IMS proteins [9]. Bax and Bak are clearly involved in the formation of such OMM pores or channels; the oligomerization of these pro-apoptotic proteins somehow engages mitochondria into apoptotic signaling [10], [11]. The nature of these pores is subject of much debate, with consideration of both proteinaceous or lipidic pores [12], [13].

Despite the apparent non-specific nature of the OMM pores, even in intact cells, the redistribution of IMS proteins does not necessarily occur simultaneously, but may indeed occur differentially across the permeabilized OMM. Some studies showed that cyt c and Smac were redistributed simultaneously during apoptosis, while others reported that release of cyt c occurred prior to that of Smac [14], [15], [16], [17]. These diverse observations may arise from the study of different cell types, the nature of apoptotic inducers and the particular techniques employed in each study. Moreover, mechanistic factors may be involved including tethering of IMS proteins (such as cyt c or Smac) in the IMS, which can contribute to a delay in the release of an individual protein through permeabilized OMM [18], [19], [20].

From the analytical perspective, sub-cellular fractionation followed by western blotting has been commonly used to study the redistribution of individual IMS proteins. However, this procedure determines the overall redistribution of proteins in whole cell populations rather than in individual cells. Immunocytochemical techniques offer the advantage of monitoring redistribution of cyt c and Smac to the cytosol at a single cell level by applying two antibodies simultaneously [17]. This method has appropriate definition of potentially differential redistribution, but involves manual scoring of many fields of cells, which can be laborious.

Accordingly, we developed a high throughput technique based on flow cytometry to analyze redistribution of cyt c and Smac simultaneously from mitochondria during apoptotic signaling. Flow cytometry was initially applied by Waterhouse et al. [21] to study the release of cyt c as a “solo” protein from mitochondria, in which cells were treated with digitonin to permeabilize the plasma membrane followed by application of primary and secondary antibodies. In this report, we first describe how we set up the duplexed version of the flow cytometry procedure. This was initially applied to the 143B TK^-^ cell line (hereinafter called 143B) treated with staurosporine (STS), in which it was previously shown that redistribution of cyt c from mitochondria preceded that of Smac [17]. We went on to apply the duplexed flow cytometry to other cells, under various conditions and validated data by the more tedious immunocytochemistry method. We found that the redistribution of cyt c and Smac is reproducibly concurrent or differential, depending on cell line and apoptotic inducer.

## Results and Discussion

### Principle of the Flow Cytometry Duplexed System

**Figure 1:**
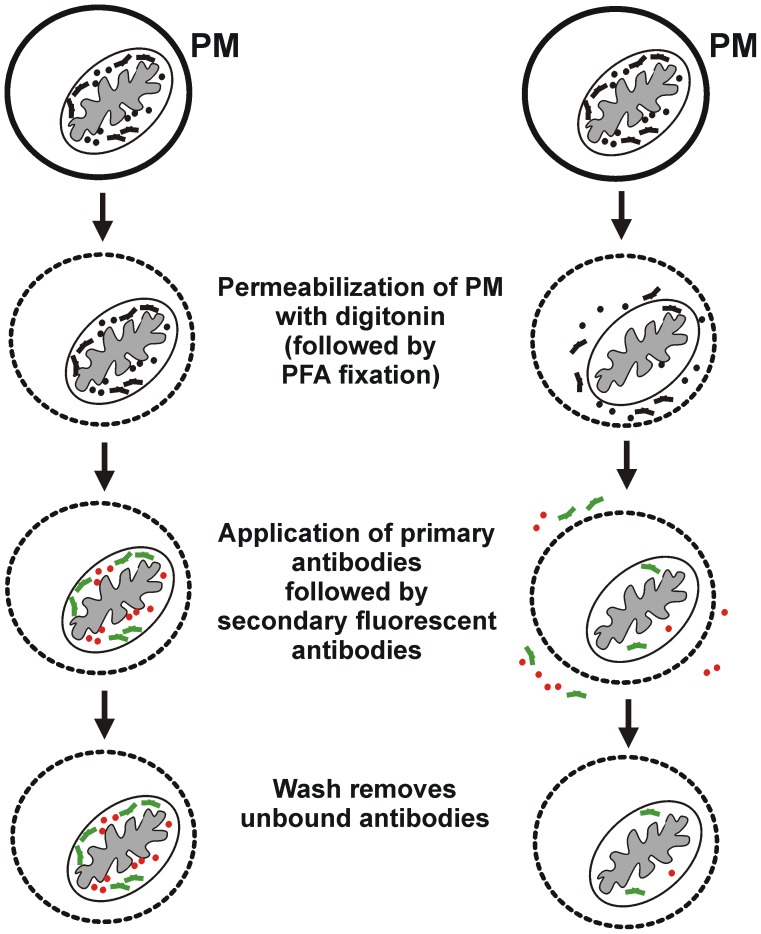
Schematic of digitonin permeabilization of plasma membrane to detect redistribution of cyt c and Smac by flow cytometry. Cells are permeabilized with digitonin followed by paraformaldehyde (PFA) fixation. Primary antibodies specific for target proteins cyt c or Smac, and fluorescently-tagged secondary antibodies, are applied to the cells followed by washing steps to remove unbound antibodies. (Left side) Flow cytometry detects cells that retain cyt c (red) or Smac (green) in mitochondria through the binding of fluorescently-tagged antibodies. (Right side) Cells that have redistributed either cyt c or Smac to the cytosol lose almost all of each protein through the permeabilized plasma membrane (PM) (dotted line).

In principle, retention of cyt c and Smac in mitochondria allows the differentially fluorescently-tagged secondary antibodies to bind and thus be detected by flow cytometry (Figure 1; left side). However, cells in which cyt c or Smac have redistributed to the cytosol will undergo dispersion of either one or both of those proteins through the digitonin-permeabilized plasma membrane. Consequently, the proteins will not be detected by flow cytometry (Figure 1; right side).

**Figure 2:**
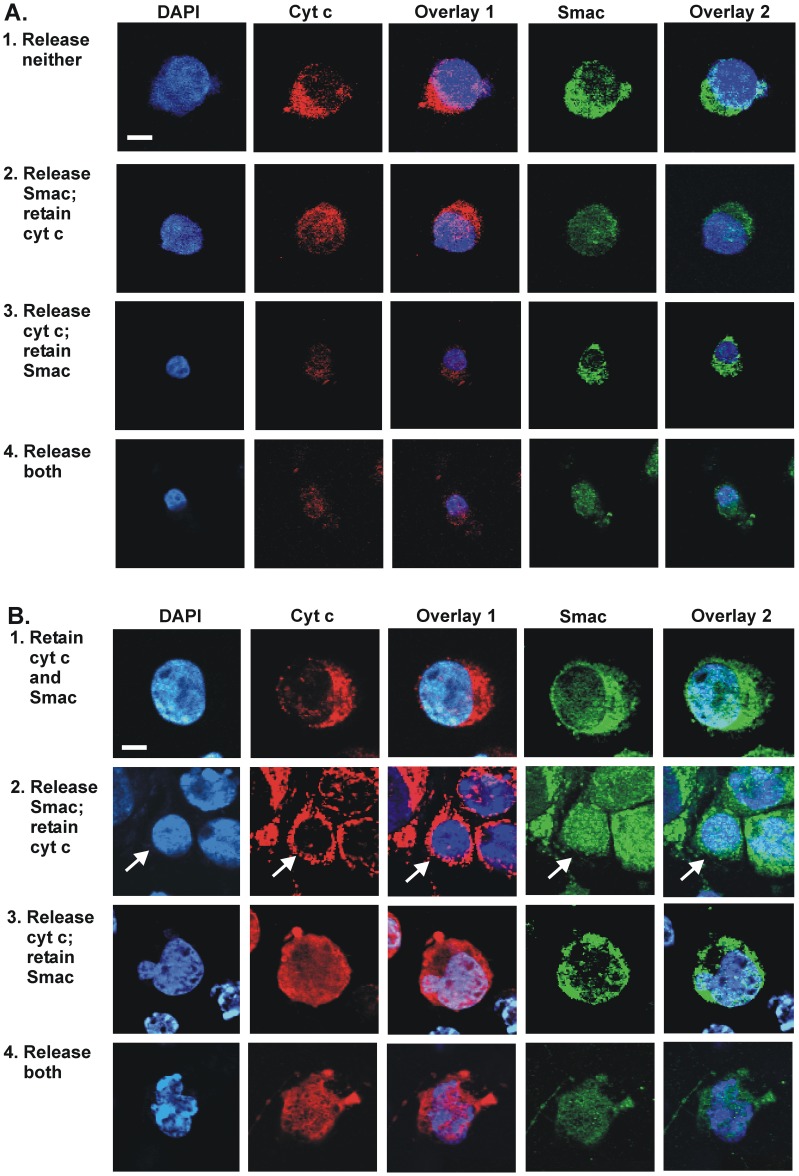
Confocal microscopic imaging of single cells. All images shown are from cultures of 143B cells treated with STS for 24 h. (A) Representative images of cells permeabilized with digitonin, showing retention or redistribution of cyt c or Smac from mitochondria. Cells were processed as for flow cytometry, then immunostained with antibodies specific for cyt c and Smac. Cells were then stained with DAPI (to visualize the nucleus) and finally centrifuged on coverslips prior to confocal microscopy. Images were acquired by confocal microscopy using a FluoView 500 with 60x objective lens. Overlay 1 is a merge between DAPI stain (blue channel) and cyt c (red channel); Overlay 2 is a merge between DAPI stain (blue channel) and Smac (green channel). (B) Representative images of 143B cells subjected to direct immunocytochemical procedures with antibodies specific for cyt c and Smac, and also DAPI-stained, showing retention or redistribution of cyt c or Smac. Cells were grown in 6-well plates, then centrifuged to recover both detached and adherent cells, which were then fixed with 3.5% paraformaldehyde and treated with Triton X-100 prior to immunostaining. Slightly different nomenclature is used to describe the complementary attributes of the top rows of cells in (A) and (B) because flow cytometry detects cells that retain the target protein while direct immunocytochemistry scoring is based on the observed redistribution of target proteins from mitochondria to the cytosol (but still within the boundary plasma membrane). Arrow indicates relevant cell in Row B2. Scale bar (20 µm) applies to all images in each of panels A and B. Very occasionally, significant amounts of Smac may appear in nucleus (cf. ref [29]), but this is extremely rare, with less than 3% of cells showing such localization of Smac (data not shown).

We applied confocal microscopy to support the principle that cyt c and Smac, after redistribution from mitochondria, were dispersed completely from digitonin-permeabilized cells. These confocal microscopy images (Figure 2A) validated our approach through the visualization of the subcellular location of cyt c and Smac in cells subjected to digitonin-permeabilization. For comparison, direct immunocytochemical analysis of non-digitonin-permeabilized cells was carried out (Figure 2B). In the latter case, cells were fixed and permeabilized with Triton X-100. DAPI staining of the nucleus (blue channel) indicates the location of a particular cell of interest in the population.

In the top row of Figure 2A, a non-STS treated cell retains cyt c and Smac in mitochondria, with clearly discernible DAPI stain (overlay 1 and 2 in top row of Figure 2A). This retention feature is represented by the punctate staining in red and green channels for cyt c and Smac, respectively. In the case of such digitonin-permeabilized cells undergoing apoptosis, the grossly diminished fluorescence staining of cyt c or Smac (Figure 2A, rows 2–4), indicates the loss of redistributed cyt c or Smac to the cytosol. This validates the principle of flow cytometry to detect preferential redistribution of mitochondrial proteins.

The loss of cyt c or Smac, or both, following redistribution from mitochondria in digitonin-permeabilized cells (Figure 2A) is to be contrasted with the fluorescence images of fixed cells directly subjected to immunocytochemistry (Figure 2B, rows 2–4). The redistributed protein in each case remains dispersed within the cell; fluorescence is clearly discernible across the cytoplasmic area as well as the nuclear region (refer overlays 1 and 2 in Figure 2B). Note that cells not exposed to STS display clearly both cyt c and Smac in mitochondria (Figure 2B, top row).

### Quadrant Delineation in Flow Cytometry Analysis

**Figure 3:**
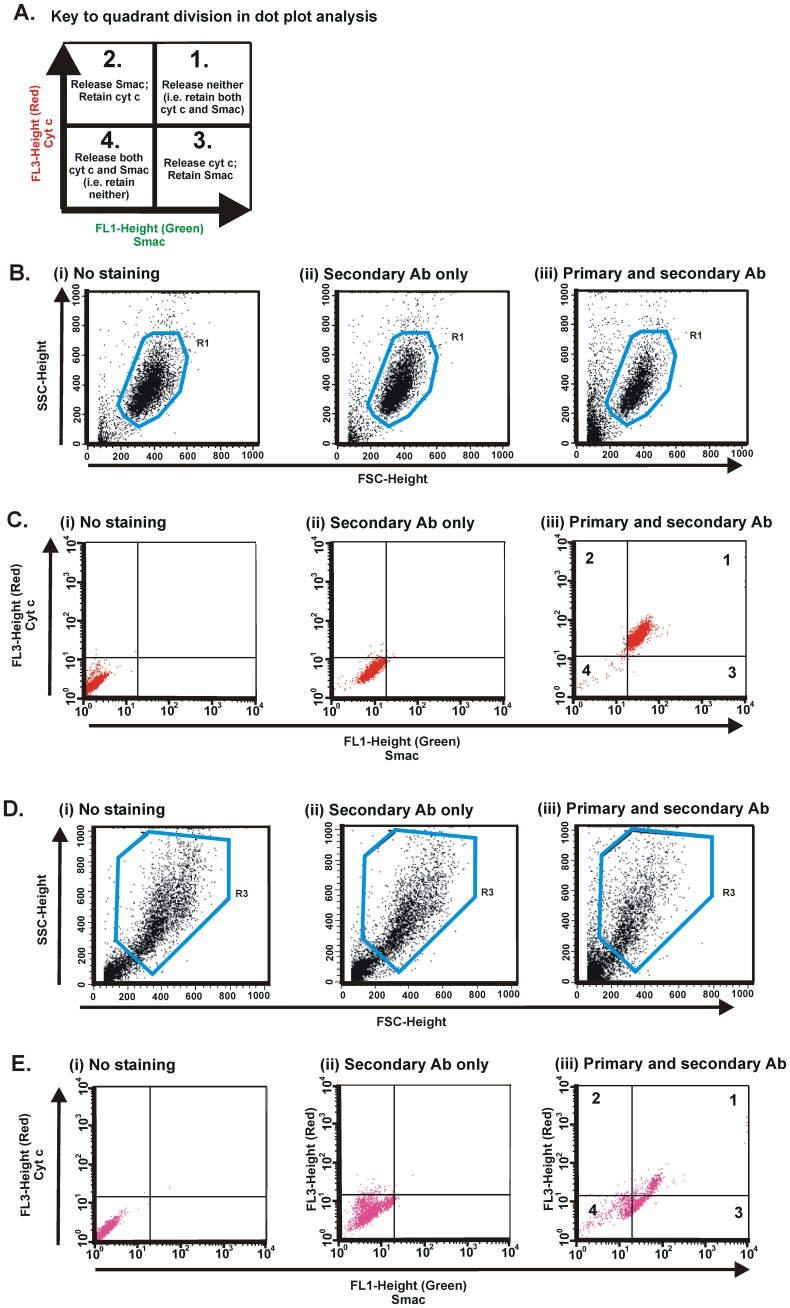
Dot plot profiles of untreated and STS-treated cells. (A) Schematic dot plot diagram showing the key to quadrant division. Each quadrant represents the apportionment of cells that retain both cyt c and Smac in mitochondria or have redistributed either or both of these proteins. (B) The populations of cells for each of the staining regimes, with the specified application of antibodies (Ab), are gated in the forward scatter (FSC) and side scatter (SSC) dot plots to eliminate dead cells and cell debris, using the “No staining” population (no antibodies applied) as the basis for this R1 gate. (C) Fluorescence dot plot analysis within the R1 gate. Populations corresponding to “No staining” (autofluorescence) and “Secondary Ab only” (non-specific binding), are located in the lower left quadrant. Cells stained with both primary and secondary antibodies are overwhelmingly located in the top right quadrant (these cells were not exposed to apoptotic inducer). Quadrant delineation is described in the text. (D) Gating of cells (R3) treated with STS for 24 h; all other indications as for panel B. Note that the R2 gate in this experiment corresponds to the 6-h time point for STS treatment, data for which are not shown here. (E) Dot plot analyses for cells treated with STS for 24 h. All indications correspond to those of panel C. Red and purple colors in panels C and E, respectively, are generated by CellQuest software (i.e. neither color refers to cyt c, specifically).

To establish the patterns of differential redistribution of cyt c and Smac from mitochondria, the data obtained from flow cytometry analysis are expressed in designated quadrants (Figure 3A). The basis of the quadrant delineation is described as follows.

Detection using flow cytometry of digitonin-permeabilized cells subjected to double-immunostaining involves examination of a population of cells in the scatter plot, by implementing a gating system or a “filter”. This enables subsequent apportionment of fluorescently-stained cells in dot plot diagrams to the appropriate quadrant. Initially, however, all cells passing through the detector are recorded in terms of their forward scatter and side scatter characteristics, which are based on the cellular complexity and size of the cells, respectively. These features allow one to set the “R1 gate” that filters out non-viable cells or cell debris (Figure 3B).

In practice, the R1 gating of viable sub-population of untreated cells is achieved by drawing a region enclosing a boundary for cells not exposed to antibodies (Figure 3B(i)). This same R1 region is applied to dot plots of untreated cells exposed only to secondary antibodies (Figure 3B(ii)) and to those exposed to both primary and secondary antibodies (Figure 3B(iii)), i.e. double-immunostained for both cyt c and Smac. The gated sub-populations for each of the samples are subsequently expressed as fluorescence dot plot analyses with FL1 (x-axis) and FL3 (y-axis) (Figure 3C).

The quadrant boundaries are set based on the quantified fluorescence emitted by cells labeled only with secondary antibodies (Figure 3C(ii)). The exact position of this quadrant in the dot plot display is then applied to fluorescence dot plot displays of fully double-immunostained samples, enabling the quadrant statistic to be generated to determine the percentage of cells in each quadrant. The vast majority of cells in the population not exposed to STS (untreated) retain both cyt c and Smac (Quadrant 1), as expected.

To characterize cells exposed to an apoptotic inducer such as STS, a new gate (e.g. R3, as in Figure 3D) must be set for each time point studied. In the example shown (24 h of treatment with STS at 100 nM), there are fewer cells within the R3 gate compared to R1 in Figure 3B, because the proportion of dead cells or cells debris is greater due to STS treatment. As above, the quadrant boundaries are re-applied based on cells labeled only with secondary antibodies (Figure 3E(ii)). The movement of a cell into a different quadrant on the fluorescence dot plot (Figure 3E(iii)) signifies redistribution from mitochondria of one or other protein, resulting from STS treatment. Here, a significant proportion of cells which have lost cyt c but not Smac through the permeabilized OMM is found in the Quadrant 3. The cells found in the Quadrant 4 have lost both cyt c and Smac. On the contrary, there are barely any cells that have lost Smac but retain cyt c (Quadrant 2).

### Quantitative Data of Flow Cytometry Analysis: cyt c Redistributes Prior to Smac in STS-treated 143B cells but not HeLa cells

**Figure 4:**
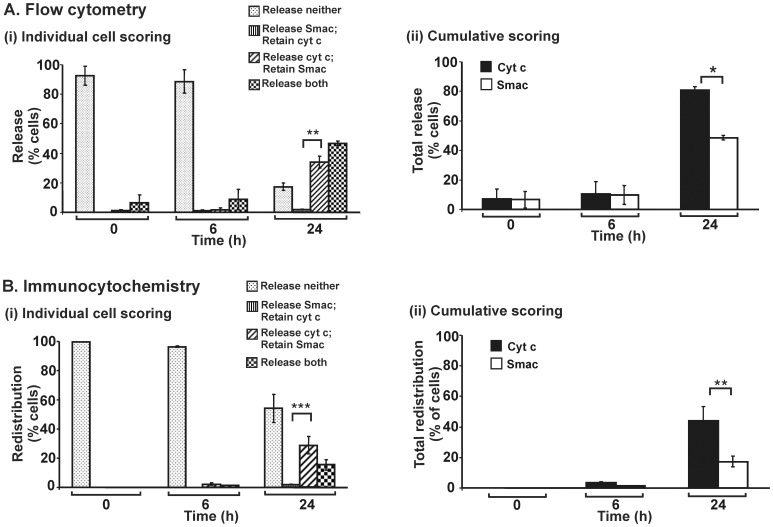
Differential redistribution of cyt c and Smac in 143B cells treated with STS. 143B cells were untreated or treated with STS (100 nM) for various times. (A) Flow cytometry data corresponding to the quantified quadrant occupancy in Figure 3C and F, averaged over three independent experiments: (i) indicates individual cell scoring for cells in each of four categories; (ii) indicates cumulative scoring for each of cyt c and Smac. Data for 3,000 events were collected for flow cytometry analysis in each individual experiment at each time point. (B) Direct immunocytochemical analyses of cells under four categories as indicated for each time point. 300 cells were scored for immunocytochemical analysis for each time point in each individual experiment. (i) and (ii) are as above for Panel A. Each standard error bar represents ± SEM from three independent experiments. Asterisks indicate significant differences of cyt c and Smac redistribution (*P<0.05; **P<0.005; ***P<0.0005).

The quadrant statistic generated from flow cytometry analysis is presented in the form of histograms (Figure 4A). Two formats of data are displayed: (i) individual cell scoring for various categories of retention or redistribution of cyt c and Smac; and (ii) cumulative scoring for each of the two proteins. Following STS exposure for 24 h, individual cell scoring data indicate that there is a significant proportion of cells that have redistributed cyt c but retained Smac. Cumulative scoring data in Figure 4A(ii) were obtained arithmetically by summing relevant data subsets from Figure 4A(i). The data show that overall, redistribution of cyt c precedes that of Smac. This confirms our previous finding that in 143B cells treated with STS, such asymmetric redistribution of cyt c and Smac occurs [17]. In the present work, we also carried out immunocytochemical analyses on STS-treated 143B cells, data for which (Figure 4B) are fully consistent with those previously reported [17], further supporting our observations based on flow cytometry.

In strictly quantitative terms, the direct immunocytochemistry method (Figure 4B) appears to underestimate the frequency of cells in which cyt c and Smac are redistributed relative to the flow cytometry approach (Figure 4A). The observer applying the direct immunocytochemistry method needs to decide, for any given cell, whether redistribution has occurred before scoring the cell. This may lead to a bias in scoring cells as retaining a given protein rather than scoring it as redistributed. On the other hand, flow cytometry may lead to overestimates of cells having redistributed a given protein, if the quadrant boundary threshold is set too low. In spite of the quantitative discrepancy, the tendency of 143B cells after STS treatment to redistribute cyt c before Smac is clearly observed by both techniques.

**Figure 5:**
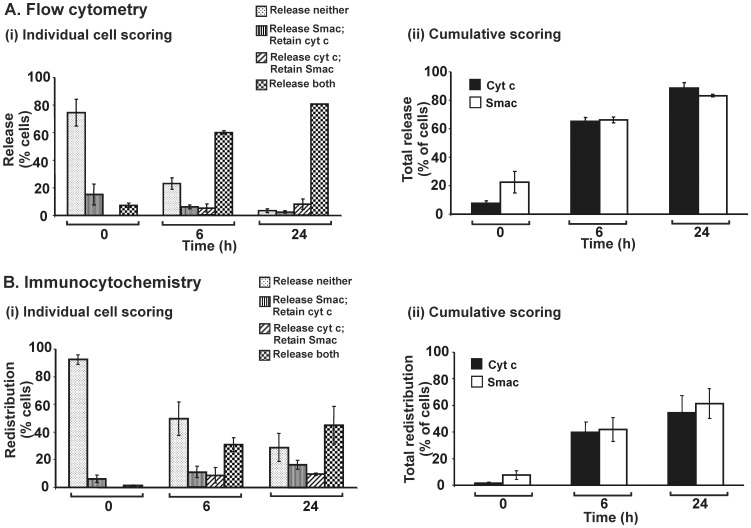
Simultaneous redistribution of cyt c and Smac in HeLa cells treated with STS. HeLa cells were untreated or treated with STS (300 nM) for various times. All other indications as for Figure 4.

Such differential redistribution for STS-treated 143B cells is not an artefact of observation. In HeLa cells treated with STS and analyzed by the same techniques as applied above for 143B cells, the symmetrical redistribution of cyt c and Smac from mitochondria is clearly observed (Figure 5). No preferential redistribution of either protein can be seen, although flow cytometry indicates a quantitatively greater extent of redistribution than does direct immunocytochemistry.

### Effect of MT-21: Release of Smac Occurs Prior to cyt c in 143B Cells

**Figure 6:**
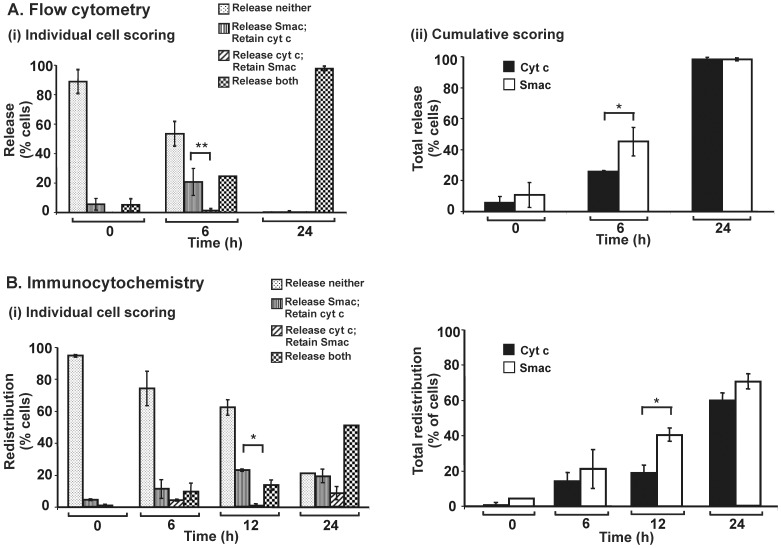
Early redistribution of Smac relative to cyt c in 143B cells treated with MT-21. 143B cells were untreated or treated with MT-21 (200 µM) for various times. All other indications as for Figure 4.

In a survey we carried out (not detailed here) of the variety of possible patterns of differential redistribution, we also found that 143B cells treated with MT-21, an apoptotic inducer that reportedly binds to the adenine nucleotide transporter of the mitochondrial inner membrane [22], [23], the differential redistribution is shifted towards Smac emerging from mitochondria earlier than cyt c. This asymmetry was evident in both flow cytometry and direct immunocytochemistry (Figure 6), being statistically significant for flow cytometry data at 6 h of MT-21 treatment, and at 12 h for immunocytochemistry. Together, these findings demonstrate and validate the power of the flow cytometry method described here to reliably quantify redistribution of mitochondria proteins to the cytosol during apoptotic signaling.

### General Perspectives

We found that redistribution of cyt c and Smac occurs differentially under various apoptotic inductions in different cell types. This pattern of preferential redistribution of cyt c or Smac suggests the presence of constraints on availability of proteins in the IMS to exit through the permeabilized OMM. Whilst in vitro studies have demonstrated that Bax can form a pore to allow passage of a molecule up to 2,000 kDa [24], OMM permeabilization is necessary but not sufficient for redistribution of a given IMS protein. In consideration of the relatively small IMS proteins analyzed here (cyt c at 12 kDa; Smac as monomer 25 kDa, but probably dimerized at 50 kDa [25]), free passage of each clearly does not necessarily occur, as evidenced here by 143B cells treated with STS or MT-21.

Therefore, there are constraints within IMS on the availability of cyt c or Smac, which must be overcome prior to their exit through the permeabilized OMM. Previous studies have shown that cyt c is bound to cardiolipin in the outer surface of the inner mitochondrial membrane. Cardiolipin is an anionic phospholipid that is present predominantly in the inner membrane, where its fluidity and stability play important roles in mitochondrial membrane structure [26]. Electrostatic interactions between cardiolipin and tethered cyt c must be disrupted to free cyt c into the IMS [18], [27]. It has further been suggested that reorganization of mitochondrial cristae (possibly modulated by the BH3-only protein tBid) is required to completely mobilize sequestered cyt c from between cristae compartments [28]. In the case of Smac, its redistribution might be modulated by mitochondrially-localized Survivin [19].

The flow cytometry procedure reported here is applicable to most cellular situations involving suspension culture or adherent cells. In the latter case, trypsinization can be used to generate cellular suspensions suitable for flow cytometry (as applied here). However, for some cell cultures (e.g. primary neurons), trypsinization often leads to cell death (anoikis) and application of the flow cytometry procedure to monitor redistribution of mitochondrial proteins such as cyt c and Smac may not be possible.

We have described here the duplexed flow cytometry procedure applied to cyt c and Smac. This procedure can be readily applied to other pairs of apoptogenic proteins redistributed from mitochondria.

## Materials and Methods

### Cell Culture

143B TK^-^ cells (ATCC catalog no. CRL-8303) and HeLa cells (ATCC catalog no. CCL-2) were cultured in complete Dulbecco’s modified Eagle’s medium (DMEM) (Invitrogen, USA) supplemented with 10% fetal bovine serum (Thermo Fisher Scientific, USA). Cells were grown as a monolayer in a 5% CO_2_ incubator at 37°C and passaged twice a week. Briefly, 143B cells were washed with 1× PBS twice and incubated with TrypLE solution (Invitrogen, USA) for 5 min at 37°C to detach cells from the culture dish by trypsinization. TrypLE solution was neutralized by adding complete DMEM medium. Cells were collected by centrifugation at 1,000 rpm for 5 min. The supernatant was discarded and the pellet was resuspended in complete DMEM medium. Prior to induction of apoptosis, cells were grown in six-well plates. Following cell counting using a hemocytometer, 2×10^5^ cells were dispensed in each well of six-well plates.

### Induction of Apoptosis

Cells were treated with 100 nM or 300 nM STS (Sigma-Aldrich, USA) for 143B and HeLa cells, respectively. MT-21 (Calbiochem, USA) was used at 200 µM to induce apoptosis in 143B cells.

### Immunofluorescence Staining of Cells Prior to Flow Cytometry Analysis

Following apoptotic induction (or for untreated control), cells were removed from six-well plates by trypsinization, as above. Both adherent and non-adherent cells were collected to represent the total cell population at each particular time point. Cells were resuspended in medium and untreated samples were counted. Similar cell counts were imputed for treated samples prior to immunofluorescence staining as described below.

1×10^5^ cells were dispensed into each well of a v-bottom 96-well plate and pelleted by centrifugation for 5 min at 1,100 rpm using a plate centrifuge. Three staining conditions were set up, each in triplicate for all cell samples (untreated or treated) as follows: “No staining”, “Secondary antibodies only” and “Primary and secondary antibodies”. Note that centrifugation was carried out to pellet down cells after each step that involves washing or incubation with blocking buffer or antibodies; the supernatant was then discarded following centrifugation. Cells were initially resuspended thoroughly in 100 µl digitonin lysis buffer (50 µg/ml digitonin; 100 mM KCl; in 1× PBS) by pipeting the cells up and down followed by incubation for 5 min at room temperature to permeabilize the plasma membrane. This concentration of digitonin (50 µg/ml) is that previously applied to 143B and HeLa cells [21]. This was followed by fixing the cells with 100 µl of 3.5% paraformaldehyde (PFA) at room temperature for 30 min. Centrifugation was carried out to remove PFA and cells were washed once with 100 µl 1× PBS. Cells were then incubated with 100 µl blocking buffer (3% bovine serum albumin; 0.05% saponin; in 1× PBS) for 30 min at room temperature. Two primary antibodies, mouse anti-cytochrome c (BD Pharmingen, USA) and rat anti-Smac/DIABLO (Calbiochem, USA), were diluted 1∶200 and 1∶100, respectively, in blocking buffer and incubated with cells overnight at 4°C. Cells were washed twice with 1× PBS. Two secondary antibodies, anti-mouse Spectral Red (Santa Cruz Biotechnology, USA) for cyt c and anti-rat Alexa 488 (Invitrogen, USA) for Smac, were diluted 1∶200, for both secondary antibodies, in blocking buffer and incubated overnight at 4°C. Cells were washed twice with 1× PBS. Cells were resuspended in blocking buffer and samples in triplicate wells were combined in the flow cytometry tube, to ensure that sufficient cells for each staining condition could be analyzed by flow cytometry. The final volume in the tube was adjusted to 400 µl with blocking buffer.

Optimization of this prior immunostaining has been found to be critical for subsequent successful duplexed flow cytometry. Therefore, a detailed step-by-step protocol for preparation of cells and immunostaining, enabling subsequent quantification of redistribution of cyt c and Smac from mitochondria using flow cytometry, is available as supporting material (Protocol S1).

### Flow Cytometry Detection of Redistribution of cyt c and Smac from Mitochondria

Cells were analyzed using a Becton Dickinson (San Jose, CA, USA) FACSCalibur with 488 nm laser line. A template was set up and used for each data acquisition to collect 3,000 events per sample. Fluorescence outputs for Smac (Alexa 488) and cyt c (Spectral Red) were detected in FL1 and FL3, respectively. CellQuest software (BD Biosciences, USA) was used for analysis of flow cytometry data.

### Immunocytochemical Analysis

Following apoptotic induction (or for untreated controls), both adherent and non-adherent cells were collected and centrifuged onto coverslips, as described [17]. Cells were fixed with 3.5% PFA for 10 minutes at 37°C, followed by permeabilization with 0.1% Triton-X100 at room temperature for 1 minutes. Cells were incubated in 3% BSA to block non-specific binding. Immunostaining procedures were carried out by first applying rat anti-Smac/DIABLO antibody (1∶100 dilution) and anti-rat Alexa 488 (1∶500 dilution), followed by anti-mouse cytochrome *c* antibody (1∶500 dilution) and anti-mouse Alexa 568 (Invitrogen, USA). Cells were counterstained with DAPI (0.1 µg/ml) (Invitrogen, USA) and coverslips were mounted on the slides.

### Confocal Microscopy

Images of immunofluorescent cells were acquired using Olympus FluoView 500 confocal microscope, equipped with FluoView500 software for image acquisition using UPLAN APO 60x/1.20 water immersion objective lens. Argon 488 and HeNe G 543 laser lines were used to excite Alexa Fluor 488 and Alexa Fluor 568, respectively. The emission filters BA505–525 and BA560IF were used to capture Alexa Fluor 488 and 568, respectively. DAPI stain was excited using LD405 laser line with emission filter BA430–460. In order to visualize cyt c and Smac simultaneously in multiple fluorescence channel imaging, photomultiplier sensitivities (PMT) levels were adjusted to ensure the background fluorescence levels were minimal. These optimized settings were used to image control untreated as well as treated cells.

### Statistical Analysis

All data are expressed as the mean ± standard deviation (SD) values. The data were analyzed for statistical significance by two-way ANOVA followed by Bonferroni post tests (GraphPad Prism, USA). The differences were considered significant at *p*<0.05.

## Supporting Information

Protocol S1 **Detailed protocol for preparation and immunostaining of cells for subsequent analysis by flow cytometry to determine simultaneously redistribution of cytochrome c and Smac/DIABLO from mitochondria during cell death signaling.**
(DOC)
