# Supplementary material for: Application of Flow Cytometry to Determine Differential Redistribution of Cytochrome c and Smac/DIABLO from Mitochondria during Cell Death Signaling
Source: PLoS One. 2012 Jul 27;7(7):e42298. doi: 10.1371/journal.pone.0042298 (PMC3407092; doi:10.1371/journal.pone.0042298)
Supplement: Protocol S1 — Detailed protocol for preparation and immunostaining of cells for subsequent analysis by flow cytometry to determine simultaneously redistribution of cytochrome c and Smac/DIABLO from mitochondria during cell death signaling. (DOC) [file pone.0042298.s001.doc]

**Preparation and immunostaining of cells for subsequent analysis by flow cytometry to determine simultaneously redistribution of cytochrome c and Smac/DIABLO from mitochondria during cell death signaling**

GUIDE TO SYMBOLS

* HINT

**→** ATTENTION

REAGENTS

Dulbecco’s modified eagle medium (DMEM) (Invitrogen, Catalog # 21063-045)

Fetal bovine serum (FBS) (Thermo Scientific, Catalog #15-010-0500v)

TrypLE^TM^ express stable trypsin-like enzyme with phenol red (Invitrogen, Catalog # 12605028), supplied as solution ready to use

Mouse cytochrome *c* antibody (BD Pharmingen, Catalog # 556432)

Rat anti-Smac/DIABLO antibody (Calbiochem, Catalog # 567360)

Anti Mouse Spectral Red (Santa Cruz, Catalog # 1032-13)

Anti Rat Alexa 488 (Invitrogen, Catalog # A11006)

Staurosporine (Sigma, Catalog # S4400)

Paraformaldehyde (PFA) (Sigma, Catalog # P6148-500G)

Digitonin (Sigma, Catalog # D141)

Bovine serum albumin fraction V (BSA) (Roche, Catalog # 10735078001)

Saponin (ICN Biomedicals, Catalog # 102855)

MATERIALS

Flow cytometry tubes, 5 ml polystyrene, round bottom, 12x75 mm (Falcon Becton, Dickinson and Company, Catalog #352054)

Multichannel pipette (Eppendorf)

v-bottom 96-well plate (BD Biosciences, Catalog # 353263)

PROCEDURE

Notes:

1. Protocol is devised for adherent mammalian cells. Specific conditions are described for 143BTK^-^ human osteosarcoma cells (ATCC catalog # CRL-8303). Critical steps are at immunofluorescence staining stages (particularly steps 19 and 25 in relation to the times of incubation). See Troubleshooting notes below.
2. The procedures below describe treatment of cells with staurosporine (STS) at 100 nM to induce apoptosis. Other relevant drugs can be substituted for STS.
3. All procedures are carried out at room temperature (approximately 20-24°C) except as indicated

SEEDING CELLS FOR APOPTOTIC INDUCTION

1. Remove DMEM media from 10 cm culture plates on which adherent cells have been cultured. *
2. Rinse cells twice with 1xPBS.
3. Add 2.5 ml of TrypLE to cover the culture plate.
4. Incubate in the 37°C incubator for 5 min to allow cells to detach from the plate.
5. Add 2.5 ml complete DMEM media into the culture plate, tapping dish gently on the side by hand to ensure cells have dislodged.
6. Transfer suspension of cells into 10 ml tube.
7. Centrifuge at 1,000 rpm for 5 min. Remove supernatant.
8. Resuspend cell pellet in complete DMEM medium (5 ml).
9. Count cells in suspension using hemocytometer.
10. Seed 2x10^5^ cells per well in the six-well plates.

**→** Allow triplicate wells for each time point during drug treatment, including untreated control cells containing DMEM only (without STS). For convenience, these untreated cells are to be incubated for the same time as the shortest drug treatment, at step 14.

1. Incubate plates overnight in 37°C incubator (during which time cells attach to plates).

* HINT: It is important to obtain two 10 cm culture plates, which show at least 90% confluent growth, to ensure sufficient cells are available for subsequent plating into six-well plates.

INDUCTION OF APOPTOSIS (WITH STAUROSPORINE AT 100 nM)

1. Stock STS (500 μM) is diluted in complete DMEM media to a final desired concentration (in this case, applying serial dilutions to attain 100 nM).
2. Remove medium from each well in the six-well plate.
3. Incubate cells at 37°C for desired time (e.g. 6 and 24 h).

COLLECTING CELLS READY FOR IMMUNOFLUORESCENCE STAINING

1. At the end of each treatment, medium (that may contain detached cells) is collected from each well in the six-well plate and pooled in a 15 ml tube for retention (approximately 6 ml). The remaining adherent cells are subjected to trypsinization exactly as for steps 2-5 above, then pooling this suspension together with the retained suspension containing initially detached cells. The pooled suspension is centrifuged and cell numbers counted as for steps 7-9 above. *
2. Dispense 1x10^5^ cells per well in v-bottom 96-well plate.
   Wells for three conditions (defined below) are set up, each in triplicate, for all cell samples (nine wells in total), either untreated or treated, applying this for each pooled sample at step 15.

**→** Details of conditions to be labeled on the paper template of 96-well plate are as follows:

- 1. Not exposed to either primary or secondary antibodies (no staining)
  2. Staining with both secondary antibodies only (double negative)
  3. Staining with both primary and secondary antibodies (double positive)

* HINT : Cell counting is carried out only on untreated cell populations (since treated populations may contain cell fragments and debris that may interfere with counting in the hemocytometer). The cell density in these untreated cells is then used as a guide for subsequent dispensing of all cell samples in that batch, untreated or treated at step 16.

IMMUNOFLUORESCENCE STAINING

1. Centrifuge v-bottom 96-well plate at 1,100 rpm for 5 min to pellet cells.
2. Remove supernatant from each well without disturbing the pellet. *
3. Add 100 μl of digitonin lysis buffer to each well, resuspend cells thoroughly by pipeting up and down, then incubate at room temperature for 5 min.
4. Centrifuge plate at 1,100 rpm for 5 min. Remove supernatant.
5. Add 100 μl of 3.5% of PFA, mix well, then incubate at room temperature for 30 min to fix cells.
6. Centrifuge plate at 1,100 rpm for 5 min. Remove supernatant.
7. Add 100 μl of 1x PBS to wash cells, mix well.
8. Centrifuge plate at 1,100 rpm for 5 min. Remove supernatant.
9. Suspend cells in 100 μl of blocking buffer, mix well, then incubate for 30 min at room temperature. **
10. Centrifuge plate at 1,100 rpm for 5 min. Remove supernatant. ***
11. Add 100 μl of diluted primary antibodies in blocking buffer (Mouse anti-cyt c antibody 1:200; Rat anti-Smac antibody 1:100), then incubate at 4ºC overnight.
12. Centrifuge plate at 1,100 rpm for 5 min. Remove supernatant.
13. Add 100 μl of 1x PBS to wash cells, mix well.
14. Centrifuge plate at 1,100 rpm for 5 min. Remove supernatant.
15. Repeat steps 29 and 30.
16. Add 100 μl of diluted secondary antibodies in blocking buffer (Anti-mouse Spectral red 1:150; Anti-rat Alexa 488 (green) 1:150), mix well, then incubate at 4ºC overnight.
17. Centrifuge plate at 1,100 rpm for 5 min. Remove supernatant.
18. Add 100 μl of 1x PBS to wash cells, mix well.
19. Centrifuge at 1,100 rpm for 5 min. Remove supernatant.
20. Repeat steps 34 and 35.
21. Finally, resuspend pellet in 100 μl of blocking buffer and combine all triplicate samples for each condition into a flow cytometry tube. Adjust the volume to 400 μl using blocking buffer for flow cytometry analysis.

* HINT : Use multichannel pipette for each of the relevant steps (18-37) in this section; operations are carried out for each individual well, unless otherwise stated.

** HINTS :

- 1. In general, cells need at least 30 min exposure to blocking buffer to minimize non-specific binding of antibodies.
  2. For “no staining” samples (a), leave cells in blocking buffer until step 37 (omitting removal or addition of medium or reagents at steps 26-36), adjusting volume to 100 μl at this step.
  3. Untreated and 6-h treated samples can be kept at 4ºC in the blocking buffer after fixation and washing. They can be later subjected to immunofluorescence staining procedures, together with 24-h treated samples.

*** HINT : For “double negative” samples (b), leave cells in blocking buffer at step 25, (omitting removal or addition of medium or reagents at steps 26-31), holding until double positive cells samples (c) have reached step 31, then proceed with step 32.

Cells can now be analyzed by flow cytometry in a machine such as FACSCalibur (BD Biosciences). Information on procedures for flow cytometry is in the main body of the manuscript.

RECIPES

1. Complete DMEM: add 10% fetal bovine serum
2. Digitonin lysis buffer: 50 µg/ml digitonin; 100 mM KCl in 1x PBS
3. Blocking buffer: 3% BSA; 0.05% Saponin in 1x PBS
4. 10x phosphate buffered saline (PBS) : 1.36M NaCl; 0.026M KCl; 0.1M Na_2_HPO_4_; 0.017M KH_2_PO_4_ in 1 L of H_2_O

TROUBLESHOOTING

**PROBLEM 1:**

On flow cytometry, control untreated cells show insufficient fluorescence staining in both red channel (reporting cytochrome *c*) and green channel (reporting Smac/DIABLO), for the “double positive” condition.

Possible cause and solution

Insufficient incubation time was applied for digitonin treatment (step 19). Try increased time of incubation in range 5-10 min. Note that excessive incubation times may lead to subsequent excessive non-specific binding of antibodies, observed as unacceptably high background fluorescence (in the “double negative” staining sample). Therefore, it is also important to optimize the dilution of primary or secondary antibodies by adjusting the concentration of each antibody systematically. Also, digitonin concentrations may need to be optimized (use trypan blue exclusion as a basis for determining plasma membrane permeabilization); take care not to use excessive digitonin that would permeabilize the mitochondrial outer membrane,\ as well.

**PROBLEM 2:**

Excessively high background fluorescence on flow cytometry.

Possible cause and solution

Insufficient blocking of non-specific binding of antibodies (step 25). Try extending incubation time with blocking buffer, in range 30-60 min, and/or increase concentration of BSA, in range 3-5%. In extreme cases, try other blocking agents such as fetal bovine serum or skim milk (the latter needs to be filtered to avoid blockage of the loading port in the flow cytometry). Additionally, it is also important to optimize the dilution of primary or secondary antibodies by adjusting the concentration of each antibody systematically.

EQUIPMENT

Bench top centrifuge with microplate rotor (or type similar to Heraeus Labofuge 400, Thermo Fisher Scientific, Waltham, MA, USA)

FACSCalibur flow cytometer (BD Biosciences, San Jose, CA, USA)
